# Tapping into Permutation Symmetry for Improved Detection of *k*-Symmetric Extensions

**DOI:** 10.3390/e25101425

**Published:** 2023-10-08

**Authors:** Youning Li, Chao Zhang, Shi-Yao Hou, Zipeng Wu, Xuanran Zhu, Bei Zeng

**Affiliations:** 1 College of Science, China Agricultural University, Beijing 100080, China; liyouning@cau.edu.cn; 2 Department of Physics, The Hong Kong University of Science and Technology, Clear Water Bay, Kowloon, Hong Kong, China; 3 College of Physics and Electronic Engineering, Center for Computational Sciences, Sichuan Normal University, Chengdu 610068, China

**Keywords:** symmetric extension, irreducible representation of *su*(*n*), permutation symmetry, computational complexity

## Abstract

Symmetric extensions are essential in quantum mechanics, providing a lens through which to investigate the correlations of entangled quantum systems and to address challenges like the quantum marginal problem. Though semi-definite programming (SDP) is a recognized method for handling symmetric extensions, it struggles with computational constraints, especially due to the large real parameters in generalized qudit systems. In this study, we introduce an approach that adeptly leverages permutation symmetry. By fine-tuning the SDP problem for detecting *k*-symmetric extensions, our method markedly diminishes the searching space dimensionality and trims the number of parameters essential for positive-definiteness tests. This leads to an algorithmic enhancement, reducing the complexity from $O(d^{2k})$ to $O(k^{d^{2}})$ in the qudit *k*-symmetric extension scenario. Additionally, our approach streamlines the process of verifying the positive definiteness of the results. These advancements pave the way for deeper insights into quantum correlations, highlighting potential avenues for refined research and innovations in quantum information theory.

## 1. Introduction

In the intricate domain of quantum mechanics, symmetric extensions stand out as a cornerstone, providing a structured mathematical lens through which to explore the nature and behavior of quantum states. A bipartite state $\rho_{AB}$ is deemed symmetrically extendible if there exists a multi-partite density matrix $\rho_{A_{1}A_{2}\ldots A_{m}B_{1}B_{2}\ldots B_{n}}$ such that each of its reduced density matrices, when traced over its complements, matches $\rho_{AB}$:

(1) $${\mathrm{Tr}}_{{\left(A_{j}B_{k}\right)}^{c}}\left(\rho_{A_{1}A_{2}\ldots A_{m}B_{1}B_{2}\ldots B_{n}}\right)=\rho_{AB},\;\forall j,k.$$

Delving into the importance of symmetric extensions, they serve as a tangible framework to probe the nature of quantum entanglement, offering a means to understand the profound correlations present in entangled quantum systems [1,2,3]. Furthermore, they pave the way for addressing the quantum marginal problem, which investigates the necessary conditions under which a set of density matrices can correspond to a global state [4,5]. This problem’s universality is showcased by its resonance with the *N*-representability problem in quantum chemistry [6,7].

A common approach for identifying a *k*-extension is to cast the problem as a semi-definite programming (SDP) problem [8,9,10]. SDP, a form of convex optimization, involves minimizing a linear function subject to the constraints that the solution lies in the intersection of the cone of positive semi-definite matrices and an affine space. Given that density matrices are inherently semi-definite, SDP has found extensive application in quantum information problems [11,12]. By leveraging the properties of SDP, we can devise efficient algorithms for detecting *k*-extensions. For example, the algorithm used in QETLAB that determines if a bipartite quantum state $\rho_{AB}$ is *k*-symmetric extendible for the *d*-dimensional subsystem *B* has the form:

(2) $$\begin{array}{cc} \; & \mathrm{find}\;\tilde{\rho }\\ s.t. & \;\left\{\begin{array}{c} \tilde{\rho }⪰0\\ {\mathrm{Tr}}_{{\left(AB_{1}\right)}^{c}}\left(\tilde{\rho }\right)=\rho_{AB}\\ \left(1_{A}⊗P_{ij}\right)\tilde{\rho }\left(1_{A}⊗P_{ij}\right)=\tilde{\rho },\forall i,j \end{array}\right. \end{array}$$

where $\tilde{\rho }=\rho_{AB_{1}B_{2}\ldots B_{k}}$ is the *k*-symmetric extension of $\rho_{AB}$ and the operator $P_{ij}$ is an element in the permutation group $S_{k}$. However, the substantial number of real parameters, notably in the general qudit scenario, can present formidable computational obstacles. This is largely due to the requirement of the entire extended Hilbert space $H_{AB_{1}B_{2}\ldots B_{k}}$, which scales as $O({\left(d^{2}\right)}^{k})$. Such exponential scaling can make calculations intractable for larger systems or higher dimensions.

In this work we are going to present a new optimization scheme, which not only considers the permutation symmetry to reduce the total parameters, but also optimizes the subroutine to determine positive definiteness, where the parameters for single-time optimization grow no faster than

$$\prod_{m=1}^{d-1}\frac{1}{m!}{\left(1+\frac{2k}{d(d+1)}\right)}^{d(d-1)/2},$$

for large *k* and *d*.

A testament to our methodology’s effectiveness is its application to the renowned bipartite Werner state, where it exhibits a pronounced acceleration in comparison to the established QETLAB software. This enhancement equips us to approach larger *k*-extension challenges with unparalleled efficiency.

Furthermore, our calculations have explicitly determined the dimensions of the searching space and the number of parameters required for positive-definiteness tests. This efficiency stems from our algorithm’s ability to undergo multiple distinct positive-definiteness tests, each correlating to a unique Young diagram. Each individual test, though involving a significantly smaller matrix, culminates in a comprehensive and efficient analysis.

Our findings contribute to a clearer understanding of quantum systems, potentially aiding in the design of more proficient quantum algorithms and enhancing our grasp of quantum information theory.

The structure of our paper is as follows: Section 2 delves into the intricacies of the 3-extension of the qutrit case as an illustrative example. Section 3 elucidates our methodology to compute the reduced density matrix of global states for a general *k*-extendible state, and underscores our rationale for dimensionality reduction. Section 4 shows a comparison of our new algorithm and the traditional one. Concluding insights and discussions are furnished in Section 5.

## 2. Qutrit Example

Before starting to solve the general problem, we first take a look at two simple examples, 2- and 3-extensions of qutrits. In fact, these two cases clearly demonstrate why our new algorithm can greatly reduce the size of the searching space. We are going to investigate how many real parameters are needed to fully describe the global symmetric extended matrix $\rho_{A\vec{B}}$, which lies in Hilbert space $V=V_{A}⊗T$ constituted by parts *A* and $\vec{B}$, with the constraints that ${\mathrm{Tr}}_{{\left(AB_{1}\right)}^{c}}\left(\rho_{A\vec{B}}\right)=\rho_{AB}$.

### 2.1. 2-Qutrit Case

In this case, $T\equiv V^{\left(1\right)}⊗V^{\left(2\right)}$ is constituted by the two qutrits $B_{1}$ and $B_{2}$, where $V^{\left(1\right)}$ and $V^{\left(2\right)}$ represent $B_{1}$ and $B_{2}$, respectively. T is spanned by nine vectors {|00〉,|01〉,⋯,|22〉}. According to different permutation symmetry, T can be decomposed as a 2-invariant orthogonal subspace, a 6-dimensional bosonic one $V^{B}$, and a 3-dimensional fermionic one $V^{F}$. It is clear that there does not exist any cross term of bosonic subspace and fermionic subspace, therefore, we only have to consider the density matrix ${\bar{\rho }}_{A\vec{B}}$ supporting on $\mathrm{End}\left(V_{A}\right)⊗\mathrm{End}\left(V^{B}\right)$ and ${\tilde{\rho }}_{A\vec{B}}$ supporting on $\mathrm{End}\left(V_{A}\right)⊗\mathrm{End}\left(V^{F}\right)$.

The general form of ${\bar{\rho }}_{A\vec{B}}$ reads

(3) $${\bar{\rho }}_{A\vec{B}}=\sum_{\alpha ,\beta =1}^{6}\rho_{A}^{\left(\alpha ,\beta \right)}⊗{\bar{p}}_{\alpha ,\beta }|\phi_{\vec{B}}^{\alpha }\rangle \langle \;\phi_{\vec{B}}^{\beta }|,$$

where $\rho_{A}^{\left(\alpha ,\beta \right)}\in \mathrm{End}\left(V_{A}\right)$, ${\bar{p}}_{\alpha ,\beta }$ is a complex number and

$$\begin{array}{ccc} \left\{|\phi_{\vec{B}}^{\alpha }\rangle \right\} & = & \left\{|00\rangle ,|11\rangle ,|22\rangle ,\frac{1}{\sqrt{2}}(|01\rangle +|10\rangle ),\frac{1}{\sqrt{2}}(|02\rangle +|20\rangle ),\frac{1}{\sqrt{2}}(|12\rangle +|21\rangle ),\right\}. \end{array}$$

The reduced density matrix can be obtained by performing a partial trace over $B_{2}$

(4) $$\begin{array}{c} 2\times {\mathrm{Tr}}_{V^{\left(2\right)}}\left({\bar{\rho }}_{A\vec{B}}\right)=\sum_{a,b=0}^{2}{\bar{M}}_{ab}⊗|a\rangle \langle b|, \end{array}$$

where ${\bar{M}}_{ab}$ is given by

(5) $$\begin{array}{ccc} {\bar{M}}_{00} & = & 2{\bar{p}}_{1,1}\rho_{A}^{\left(1,1\right)}+{\bar{p}}_{4,4}\rho_{A}^{\left(4,4\right)}+{\bar{p}}_{5,5}\rho_{A}^{\left(5,5\right)},\\ {\bar{M}}_{11} & = & 2{\bar{p}}_{2,2}\rho_{A}^{\left(2,2\right)}+{\bar{p}}_{4,4}\rho_{A}^{\left(4,4\right)}+{\bar{p}}_{6,6}\rho_{A}^{\left(6,6\right)},\\ {\bar{M}}_{22} & = & 2{\bar{p}}_{3,3}\rho_{A}^{\left(3,3\right)}+{\bar{p}}_{5,5}\rho_{A}^{\left(5,5\right)}+{\bar{p}}_{6,6}\rho_{A}^{\left(6,6\right)},\\ {\bar{M}}_{01} & = & {\bar{M}}_{10}^{†}=\sqrt{2}{\bar{p}}_{4,1}\rho_{A}^{\left(4,1\right)}+\sqrt{2}{\bar{p}}_{2,4}\rho_{A}^{\left(2,4\right)}+{\bar{p}}_{6,5}\rho_{A}^{\left(6,5\right)},\\ {\bar{M}}_{02} & = & {\bar{M}}_{20}^{†}=\sqrt{2}{\bar{p}}_{5,1}\rho_{A}^{\left(5,1\right)}+\sqrt{2}{\bar{p}}_{3,5}\rho_{A}^{\left(3,5\right)}+{\bar{p}}_{6,4}\rho_{A}^{\left(6,4\right)},\\ {\bar{M}}_{12} & = & {\bar{M}}_{21}^{†}=\sqrt{2}{\bar{p}}_{6,2}\rho_{A}^{\left(6,2\right)}+\sqrt{2}{\bar{p}}_{3,6}\rho_{A}^{\left(3,6\right)}+{\bar{p}}_{5,4}\rho_{A}^{\left(5,4\right)}. \end{array}$$

It is noticed that each term of the right-hand side in Equation (4) does not contain every ${\bar{p}}_{\alpha ,\beta }$. In fact, the nonzero coefficients before ${\bar{p}}_{\alpha ,\beta }$ in the term |a〉〈b| are exactly the nonzero entries of the representation matrix $T_{\left(ab\right)}$ over this bosonic invariant subspace (You may find the representation of su(3) for this case and the following case in standard textbooks on group theory, such as [13,14]).

Similarly, one can write down the general form of ${\tilde{\rho }}_{A\vec{B}}$ supporting on $\mathrm{End}\left(V_{A}\right)⊗\mathrm{End}\left(V^{F}\right)$

(6) $${\tilde{\rho }}_{A\vec{B}}=\sum_{\alpha ,\beta =1}^{3}\rho_{A}^{\left(\alpha ,\beta \right)}⊗{\tilde{p}}_{\alpha ,\beta }|\psi_{\vec{B}}^{\alpha }\rangle \langle \psi_{\vec{B}}^{\beta }|,$$

where $\rho_{A}^{\left(\alpha ,\beta \right)}\in \mathrm{End}\left(V_{A}\right)$, ${\tilde{p}}_{\alpha ,\beta }$ is a complex number and $\left\{|\psi_{\vec{B}}^{\alpha }\rangle \right\}$ is

$$\begin{array}{c} \left\{\frac{1}{\sqrt{2}}(|01\rangle -|10\rangle ),\frac{1}{\sqrt{2}}(|02\rangle -|20\rangle ),\frac{1}{\sqrt{2}}(|12\rangle -|21\rangle ),\right\}. \end{array}$$

The reduced density matrix can be obtained by performing a partial trace over $B_{2}$

(7) $$\begin{array}{c} 2\times {\mathrm{Tr}}_{V^{\left(2\right)}}\left({\tilde{\rho }}_{A\vec{B}}\right)=\sum_{a,b=0}^{2}{\tilde{M}}_{ab}⊗|a\rangle \langle b|, \end{array}$$

where ${\tilde{M}}_{ab}$ is given by

(8) $$\begin{array}{ccc} {\tilde{M}}_{00} & = & 2{\tilde{p}}_{1,1}\rho_{A}^{\left(1,1\right)}+{\tilde{p}}_{2,2}\rho_{A}^{\left(2,2\right)},\\ {\tilde{M}}_{11} & = & {\tilde{p}}_{1,1}\rho_{A}^{\left(1,1\right)}+{\tilde{p}}_{3,3}\rho_{A}^{\left(3,3\right)},\\ {\tilde{M}}_{22} & = & {\tilde{p}}_{2,2}\rho_{A}^{\left(2,2\right)}+{\tilde{p}}_{3,3}\rho_{A}^{\left(3,3\right)},\\ {\tilde{M}}_{01} & = & {\tilde{M}}_{10}^{†}={\tilde{p}}_{3,2}\rho_{A}^{\left(3,2\right)},\\ {\tilde{M}}_{02} & = & {\tilde{M}}_{20}^{†}=-{\tilde{p}}_{3,1}\rho_{A}^{\left(3,1\right)},\\ {\tilde{M}}_{12} & = & {\tilde{M}}_{21}^{†}={\tilde{p}}_{2,1}\rho_{A}^{\left(2,1\right)}. \end{array}$$

Taking both ${\bar{\rho }}_{A\vec{B}}$ and ${\tilde{\rho }}_{A\vec{B}}$ into account, far fewer real parameters are needed: the original algorithm searches the entire Hilbert space and, thus, the number of parameters is $9^{2}d_{A}^{2}$, while usage of permutation symmetry can reduce this number to $\left(6^{2}+3^{2}\right)d_{A}^{2}$. It should be stressed that such a simplification comes from the fact that the cross terms between subspaces corresponding to different permutation symmetries are forbidden.

However, the simple usage of symmetry, such as simply symmetrizing the Gell–Mann matrices over $B_{1}$ and $B_{2}$, has to determine the positive definiteness of one matrix with dimensions $\left(6^{2}+3^{2}\right)d_{A}^{2}$. As a comparison, our method involves determining the positive definiteness of two matrices, whose dimensions are $6^{2}d_{A}^{2}$ and $3^{2}d_{A}^{2}$, respectively.

### 2.2. 3-Qutrit Case

In this case, $T\equiv V^{\left(1\right)}⊗V^{\left(2\right)}⊗V^{\left(3\right)}$ is constituted by the three qutrits $B_{1}$, $B_{2}$, and $B_{3}$, where $V^{\left(i\right)}$ represents $B_{i}$. Similar to the procedure in the previous subsection, one can decompose T as the direct sum of the subspace according to different permutation symmetries, and further more, there does not exist a cross term between subspaces corresponding to different permutation symmetries. The permutation symmetry of the 3-qutrit case is much more complicated than the 2-qutrit case. It is easy to verify that there exist a 10-dimensional bosonic subspace and a 1-dimensional fermionic subspace. Therefore, one can solve this problem by imitating the previous subsection and obtaining the constraint equations. In this situation, the dimensions of the searching space can be reduced from $27^{2}d_{A}^{2}$ to $\left(10^{2}+16^{2}+1^{2}\right)d_{A}^{2}$.

However, more room is left for simplification. According to Weyl duality, the 16-dimensional subspace can be further decomposed as two orthogonal 8-dimensional invariant subspaces $T_{1}^{\left[2,1\right]}$ and $T_{2}^{\left[2,1\right]}$, and both are loaded with an equivalent su(3)-irreducible representation, described by a two-row Young diagram [2,1]. Here, $\left[\lambda \right]\equiv \left\{\lambda_{1},\lambda_{2},\cdots ,\lambda_{n}\right\}$ is a partition of integer *k*, where all $\lambda_{i}$ are integers satisfying $\lambda_{1}\geq \lambda_{2}\geq \cdots \geq \lambda_{n}\geq 0,\sum_{i=1}^{n}\lambda_{i}=k$. Such a partition is denoted by an *n*-row Young diagram.

$T_{1}^{\left[2,1\right]}$ and $T_{2}^{\left[2,1\right]}$ are spanned by the vectors $\left\{\varphi_{\vec{B}}^{\alpha ,(1)}\right\}$ and $\left\{\varphi_{\vec{B}}^{\alpha ,(2)}\right\}$:

$$\begin{array}{ccc} & & \left\{\frac{1}{\sqrt{6}}(2|001\rangle -|010\rangle -|100\rangle ),\frac{1}{\sqrt{6}}(2|002\rangle -|020\rangle -|200\rangle ),\frac{1}{\sqrt{6}}(|011\rangle -|101\rangle -2|110\rangle ),\right.\\ & & \;\frac{1}{\sqrt{12}}(2|012\rangle -|021\rangle +2|102\rangle -|120\rangle -|201\rangle -|210\rangle ),\frac{1}{\sqrt{4}}(|021\rangle -|120\rangle +|201\rangle -|210\rangle ),\\ & & \;\left.\frac{1}{\sqrt{6}}(|022\rangle +|202\rangle -2|220\rangle ),\frac{1}{\sqrt{6}}(2|112\rangle -|121\rangle -|211\rangle ),\frac{1}{\sqrt{6}}(|122\rangle +|212\rangle -2|221\rangle )\right\}, \end{array}$$

$$\begin{array}{ccc} & & \left\{\frac{1}{\sqrt{2}}(|010\rangle -|100\rangle ),\frac{1}{\sqrt{2}}(|020\rangle -|200\rangle ),\frac{1}{\sqrt{2}}(|011\rangle -|101\rangle ),\right.\\ & & \;\frac{1}{\sqrt{4}}(|021\rangle +|120\rangle -|201\rangle -|210\rangle ),\frac{1}{\sqrt{12}}(2|012\rangle +|021\rangle -2|102\rangle -|120\rangle -|201\rangle +|210\rangle ),\\ & & \;\left.\frac{1}{\sqrt{2}}(|022\rangle -|202\rangle ),\frac{1}{\sqrt{2}}(|121\rangle -|211\rangle ),\frac{1}{\sqrt{2}}(|122\rangle -|212\rangle ),\right\}. \end{array}$$

Under the constraint condition imposed in Equation (1), the general form of global state ${\hat{\rho }}_{A\vec{B}}$ supporting on $\mathrm{End}\left(V_{A}\right)⊗\mathrm{End}\left(T_{1}^{\left[2,1\right]}⊕T_{2}^{\left[2,1\right]}\right)$ must be of the following form:

(9) $${\hat{\rho }}_{A\vec{B}}=\sum_{\alpha ,\beta =1}^{8}\rho_{A}^{\left(\alpha ,\beta \right)}⊗{\hat{p}}_{\alpha ,\beta }\left(|\varphi_{\vec{B}}^{\alpha ,(1)}\rangle \langle \varphi_{\vec{B}}^{\beta ,(1)}\left|+\right|\varphi_{\vec{B}}^{\alpha ,(2)}\rangle \langle \varphi_{\vec{B}}^{\beta ,(2)}|\right),$$

The reduced density matrix can be obtained by performing a partial trace over $B_{2}$

(10) $$\begin{array}{ccc} & & \frac{3}{2}\times {\mathrm{Tr}}_{V^{\left(2\right)}}\left({\hat{\rho }}_{A\vec{B}}\right)=\sum_{a,b=0}^{2}{\hat{M}}_{ab}⊗|a\rangle \langle b|, \end{array}$$

where ${\tilde{M}}_{ab}$ is given by

(11) $$\begin{array}{ccc} {\hat{M}}_{00} & = & 2{\hat{p}}_{1,1}\rho_{A}^{\left(1,1\right)}+2{\hat{p}}_{2,2}\rho_{A}^{\left(2,2\right)}+{\hat{p}}_{1,1}\rho_{A}^{\left(1,1\right)}+{\hat{p}}_{4,4}\rho_{A}^{\left(4,4\right)}+p_{5,5}\rho_{A}^{\left(5,5\right)}+{\hat{p}}_{6,6}\rho_{A}^{\left(6,6\right)},\\ {\hat{M}}_{11} & = & {\hat{p}}_{1,1}\rho_{A}^{\left(1,1\right)}+2{\hat{p}}_{3,3}\rho_{A}^{\left(3,3\right)}+{\hat{p}}_{4,4}\rho_{A}^{\left(4,4\right)}{\hat{p}}_{5,5}\rho_{A}^{\left(5,5\right)}+2{\hat{p}}_{7,7}\rho_{A}^{\left(7,7\right)}+{\hat{p}}_{8,8}\rho_{A}^{\left(8,8\right)},\\ {\hat{M}}_{22} & = & {\hat{p}}_{2,2}\rho_{A}^{\left(2,2\right)}+{\hat{p}}_{4,4}\rho_{A}^{\left(4,4\right)}+{\hat{p}}_{5,5}\rho_{A}^{\left(5,5\right)}+2{\hat{p}}_{6,6}\rho_{A}^{\left(6,6\right)}+{\hat{p}}_{7,7}\rho_{A}^{\left(7,7\right)}+2{\hat{p}}_{8,8}\rho_{A}^{\left(8,8\right)},\\ {\hat{M}}_{01}={\hat{M}}_{10}^{†} & = & -\frac{1}{\sqrt{2}}{\hat{p}}_{4,1}\rho_{A}^{\left(4,1\right)}+\sqrt{\frac{3}{2}}{\hat{p}}_{5,1}\rho_{A}^{\left(5,1\right)}+{\hat{p}}_{6,2}\rho_{A}^{\left(6,2\right)}+\frac{1}{\sqrt{2}}{\hat{p}}_{8,4}\rho_{A}^{\left(8,4\right)}-\sqrt{\frac{3}{2}}{\hat{p}}_{8,5}\rho_{A}^{\left(8,5\right)}-{\hat{p}}_{7,3}\rho_{A}^{\left(7,3\right)},\\ {\hat{M}}_{02}={\hat{M}}_{20}^{†} & = & {\hat{p}}_{2,1}\rho_{A}^{\left(2,1\right)}+\frac{1}{\sqrt{2}}{\hat{p}}_{4,3}\rho_{A}^{\left(4,3\right)}+\sqrt{\frac{3}{2}}{\hat{p}}_{5,3}\rho_{A}^{\left(5,3\right)}+\frac{1}{\sqrt{2}}{\hat{p}}_{6,4}\rho_{A}^{\left(6,4\right)}+\sqrt{\frac{3}{2}}{\hat{p}}_{6,5}\rho_{A}^{\left(6,5\right)}+{\hat{p}}_{2,1}\rho_{A}^{\left(2,1\right)},\\ {\hat{M}}_{12}={\hat{M}}_{21}^{†} & = & {\hat{p}}_{3,1}\rho_{A}^{\left(3,1\right)}+\sqrt{2}{\hat{p}}_{4,2}\rho_{A}^{\left(4,2\right)}+{\hat{p}}_{7,4}\rho_{A}^{\left(7,4\right)}+{\hat{p}}_{8,7}\rho_{A}^{\left(8,7\right)}. \end{array}$$

Due to the permutation requirement, the number of real parameters is less than $\left(10^{2}+8^{2}+1^{2}\right)d_{A}^{2}$, since some pairs of α and β may contribute nothing when computing the one-body reduced density matrix (but these numbers cannot be set to 0 directly, since they may affect the positive definiteness). It should be stressed that the simplification comes not only from the fact that the cross terms between subspaces corresponding to different permutation symmetries are forbidden, but also arises from the fact that the majority of the cross terms within subspaces corresponding to identical permutation symmetries are also forbidden. It should also be noticed that, via our method, one can check the positive definiteness of the global state by successively checking the positive definiteness of the density matrix corresponding to different permutation symmetries.

## 3. Complexity of Improved SDP

In this section, we are going to give the general form of a global state that corresponds to the given quantum marginals $\rho_{AB}$.

Consider the symmetric extension problem described in Equation (2). It is required that the global state $\rho_{AB_{1}\cdots B_{k}}$ is invariant under any exchange of $B_{i}$ and $B_{j}$, but it does not require that $\rho_{AB_{1}\cdots B_{k}}$ must support on a subspace with specific permutation symmetry. E.g., for a 2-symmetric extendible state, its extension can be bosonic, which supports on the symmetric subspace only, or fermionic, whose support only resides on the antisymmetric subspace, or more generally, can be a mixture of both.

Consider a Hilbert space $T={⨂}_{i=1}^{k}V^{\left(i\right)}$ constituted by the *k* qudits $B_{1},B_{2},\cdots ,B_{k}$, whose computational basis is $\left\{\Phi_{i_{1},i_{2},\cdots ,i_{k}}\equiv |i_{1},i_{2},\cdots ,i_{k}\rangle \right\}$, where $i_{1},i_{2},\cdots ,i_{k}=0,1,\cdots ,d-1$.

Each subsystem $V^{\left(i\right)}$ is invariant under SU(d) ‘rotation’, and transforms according to the *d*-dimensional fundamental irreducible representation $D^{\left[1\right]}$, which corresponds to the Young diagram [1] (this is the one block Young diagram □).

Therefore, the Lie algebra su(d), which is constituted by three series of zero-trace Hermitian matrices and describes the infinitely small rotation of SU(d), has the following matrix forms on each identical $V^{\left(j\right)}$ if we set $|i\rangle$ to be the natural basis,

$$\begin{array}{ccc} {\left(T_{mn}^{\left(1\right)}\right)}_{st} & = & \frac{1}{2}\left(\delta_{ms}\delta_{nt}+\delta_{ns}\delta_{mt}\right),\\ {\left(T_{mn}^{\left(2\right)}\right)}_{st} & = & \frac{-i}{2}\left(\delta_{ms}\delta_{nt}+\delta_{ns}\delta_{mt}\right),\\ {\left(T_{p}^{\left(3\right)}\right)}_{st} & = & \left\{\begin{array}{cc} ll\delta_{st}{\left[2\left(p+1\right)p\right]}^{-\frac{1}{2}}, & s<p,\\ -\delta_{st}{\left[p/\left(2p+2\right)\right]}^{\frac{1}{2}}, & s=p,\\ 0, & s>p, \end{array}\right., \end{array}$$

where m<n, and 1≤p≤d−1. Taking the global phase into account, one should also include the identity matrix. Therefore, one can obtain a new basis for Lie algebra u(d) by

$$\{T_{ab}|{\left(T_{ab}\right)}_{st}=\delta_{as}\delta_{bt},0\leq a,b\leq d-1\}.$$

T is also invariant under the global U(d) transformation, whose corresponding Lie algebra is given by $\left\{T_{ab}|T_{ab}=\sum_{i}T_{ab}^{\left(i\right)}\right\}$ (here, $T_{ab}^{\left(i\right)}$ denotes that the *i*-th subsystem transforms according to $T_{ab}$ while others transform according to the identity operator). T transforms under representation ${⊗}^{k}\left[1\right]$, which is not irreducible, but can be decomposed as the direct sum of a series of irreducible representations,

(12) $$\begin{array}{c} {⨂}^{k}D^{\left[1\right]}=\underset{\left[\lambda \right]}{⨁}m_{\left[\lambda \right]}D^{\left[\lambda \right]}, \end{array}$$

where $m_{\left[\lambda \right]}$ is the multiplicity of the irreducible representation $D^{\left[\lambda \right]}$. This is equivalent to saying that T can be partitioned as the direct sum of subspaces. (Please note that subspaces corresponding to different Young diagrams are orthogonal to each other, while those corresponding to the same Young diagram are not. However, it is guaranteed that the intersection of two such different subspaces is zero.)

(13) $$T=\underset{\left[\lambda \right]}{⨁}m_{\left[\lambda \right]}T^{\left[\lambda \right]}.$$

It can be easily shown that such a $T^{\left[\lambda \right]}$ has a particular permutation symmetry described by Young diagram [λ], and the multiplicity $m_{\left[\lambda \right]}$ equals the dimension of the irreducible representation of $S_{k}$ corresponding to the identical Young diagram [λ], which gives the equation

(14) $$d^{k}=\sum_{\left[\lambda \right]}m_{\left[\lambda \right]}D^{\left[\lambda \right]}.$$

Two irreducible representation spaces $T_{\mu }^{\left[\lambda \right]}$ and $T_{\nu }^{\left[\lambda \right]}$ corresponding to the same Young diagram but different Young tableaus are orthogonal to each other. Although there might probably be multiplicity in some weight subspace for a general irreducible subspace, one can uniquely label a vector within an arbitrary given irreducible subspace by its weight $\vec{\omega }$ in su(d) and the subgroup chain su(d)⊃su(d−1)⊃⋯⊃su(2) [15]. Thus, one can safely use the weight $\vec{\omega }$ to label different states inside an irreducible subspace $T_{\mu }^{\left[\lambda \right]}$. Therefore, $\left\{|\left[\lambda \right],\mu ,\vec{\omega }\rangle \right\}$ labels a complete basis of T one by one, where [λ] denotes non-equivalent su(d) representations, and μ differentiates equivalent ones. Together they determine an orthogonal irreducible subspace, and $\vec{\omega }$ labels every different vector inside.

On the other hand, $\left\{|[\lambda ],\mu ,\omega \rangle \right\}$ can be interpreted in another way: $\vec{\omega }$ describes the weight, and [λ] denotes non-equivalent $S_{k}$ representations, thus, these two parameters differentiate orthogonal invariant subspaces, while μ labels vectors inside. From now on, we shall use $|{\vec{\omega }}_{\mu }^{\left[\lambda \right]}\rangle$ as short for $|\vec{\omega },\left[\lambda \right],\mu \rangle$.

Any matrix $\rho_{AB_{1}B_{2}\cdots B_{n}}\in \mathrm{End}\left(V_{A}\right)⊗\mathrm{End}\left(T\right)$ can be expressed as

(15) $$\rho_{AB_{1}B_{2}\cdots B_{k}}=\sum_{\alpha ,\alpha^{\prime }}\sum_{\left[\lambda \right],\left[\lambda^{\prime }\right]}\sum_{\mu ,\mu^{\prime }}\sum_{\vec{\omega },\vec{\omega^{\prime }}}|\psi_{\vec{\omega },\left[\lambda \right],\mu }^{\alpha }\rangle \langle \psi_{\vec{\omega^{\prime }},\left[\lambda^{\prime }\right],\mu^{\prime }}^{\alpha^{\prime }}|⊗|{\vec{\omega }}_{\mu }^{\left[\lambda \right]}\rangle \langle {\vec{\omega^{\prime }}}_{\mu^{\prime }}^{\left[\lambda^{\prime }\right]}|,$$

where $|\psi_{\vec{\omega },\left[\lambda \right],\mu }^{\alpha }\rangle$ are non-normalized states in $V_{A}$ and α labels different states in $V_{A}$.

Inserting Equation (15) into Equation (2), $\forall \pi \in S_{k}$, we obtain a series of constraints for $\rho_{AB_{1}B_{2}\cdots B_{k}}$:

(16) $$\begin{array}{ccc} & & \;\forall \left[\lambda \right],\left[\lambda^{\prime }\right]\vec{\omega },\vec{\omega^{\prime }}\mathrm{and}\mu ,\mu^{\prime },\\ & & \sum_{\alpha ,\alpha^{\prime }}|\psi_{\vec{\omega },\left[\lambda \right],\mu }^{\alpha }\rangle \langle \psi_{\vec{\omega^{\prime }},\left[\lambda^{\prime }\right],\mu^{\prime }}^{\alpha^{\prime }}|\sum_{\nu ,\nu^{\prime }}A{\left(\pi \right)}_{\mu ,\nu }^{\left[\lambda \right]}A{\left(\pi \right)}_{\nu^{\prime },\mu^{\prime }}^{[\lambda^{\prime }]*}|{\vec{\omega }}_{\nu }^{\left[\lambda \right]}\rangle \langle {\vec{\omega^{\prime }}}_{\nu^{\prime }}^{\left[\lambda^{\prime }\right]}|\\ & & \;=\sum_{\alpha ,\alpha^{\prime }}|\psi_{\vec{\omega },\left[\lambda \right],\mu }^{\alpha }\rangle \langle \psi_{\vec{\omega^{\prime }},\left[\lambda^{\prime }\right],\mu^{\prime }}^{\alpha^{\prime }}||{\vec{\omega }}_{\mu }^{\left[\lambda \right]}\rangle \langle {\vec{\omega^{\prime }}}_{\mu^{\prime }}^{\left[\lambda^{\prime }\right]}|,\; \end{array}$$

where $A^{\left[\lambda \right]}$ and $A^{\left[\lambda^{\prime }\right]}$ are irreducible representations of the permutation group $S_{k}$.

Define matrix

(17) $$M\left(\vec{\omega },\vec{\omega^{\prime }},\left[\lambda \right],\left[\lambda^{\prime }\right]\right)\equiv \sum_{\mu ,\mu^{\prime }}M{\left(\vec{\omega },\vec{\omega^{\prime }},\left[\lambda \right],\left[\lambda^{\prime }\right]\right)}_{\mu \mu^{\prime }}|{\vec{\omega }}_{\mu }^{\left[\lambda \right]}\rangle \langle {\vec{\omega^{\prime }}}_{\mu^{\prime }}^{\left[\lambda^{\prime }\right]}|,$$

where

(18) $$M{\left(\vec{\omega },\vec{\omega^{\prime }},\left[\lambda \right],\left[\lambda^{\prime }\right]\right)}_{\mu \mu^{\prime }}\equiv \sum_{\alpha ,\alpha^{\prime }}|\psi_{\vec{\omega },\left[\lambda \right],\mu }^{\alpha }\rangle \langle \psi_{\vec{\omega^{\prime }},\left[\lambda^{\prime }\right],\mu^{\prime }}^{\alpha^{\prime }}|,$$

thus, $\forall \pi \in S_{k}$

(19) $$A^{\left[\lambda \right]}\left(\pi \right)M\left(\vec{\omega },\vec{\omega^{\prime }},\left[\lambda \right],\left[\lambda^{\prime }\right]\right)A^{\left[\lambda^{\prime }\right]}{\left(\pi \right)}^{†}=M\left(\vec{\omega },\vec{\omega^{\prime }},\left[\lambda \right],\left[\lambda^{\prime }\right]\right).$$

Schur’s lemma guarantees that,

a. when $\left[\lambda \right]\neq \left[\lambda^{\prime }\right]$, M=0;
b. when $\left[\lambda \right]=\left[\lambda^{\prime }\right]$, *M* is invertible.

$|{\vec{\omega }}_{\mu }^{\left[\lambda \right]}\rangle$ should be chosen carefully such that the representations $A^{\left[\lambda \right]}$ are identical, not just an isomorphic matrix, for different weights ω. Then, all $M(\vec{\omega },\vec{\omega^{\prime }},\left[\lambda \right],\left[\lambda \right])$ can be proportional to the corresponding identity matrix. Therefore, one can eliminate the majority of cross terms and restrict $\rho_{AB_{1}B_{2}\cdots B_{k}}$ to

(20) $$\rho_{AB_{1}B_{2}\cdots B_{k}}=\sum_{\left[\lambda \right]}\sum_{\vec{\omega },\vec{\omega^{\prime }}}f\left(\left[\lambda \right],\vec{\omega },\vec{\omega^{\prime }}\right)\sigma \left(\left[\lambda \right],\vec{\omega },\vec{\omega^{\prime }}\right)⊗\sum_{\mu }|{\vec{\omega }}_{\mu }^{\left[\lambda \right]}\rangle \langle {\vec{\omega }}_{\mu }^{\prime [\lambda ]}|,$$

where $f(\left[\lambda \right],\vec{\omega },\vec{\omega^{\prime }})$ is the coefficient and $\sigma \left(\left[\lambda \right],\vec{\omega },\vec{\omega^{\prime }}\right)\in \mathrm{End}\left(V_{A}\right)$ (this does not have to be a density matrix!), both of which correspond to the $S_{k}$-irreducible representation described by the Young diagram [λ] and different weights $\vec{\omega }$ and $\vec{\omega^{\prime }}$.

Our next task is to determine the RDM of the global state given by Equation (20). For every given $\left[\lambda \right],\vec{\omega }$, and $\vec{\omega^{\prime }}$, one can temporally ignore system *A* and concentrate on group $\left\{B_{1},B_{2},\cdots ,B_{k}\right\}$,

(21) $$\begin{array}{ccc} \sum_{i,j=0}^{d-1}B_{ij}|i\rangle \langle j| & = & {\mathrm{Tr}}_{B_{1}^{c}}\left(\sum_{\mu }|{\vec{\omega }}_{\mu }^{\left[\lambda \right]}\rangle \langle {\vec{\omega }}_{\mu }^{\prime [\lambda ]}|\right)\\ & = & \sum_{i,j=0}^{d-1}|i\rangle \langle j|\mathrm{Tr}\left(T_{ji}^{\left(1\right)}\sum_{\mu }|{\vec{\omega }}_{\mu }^{\left[\lambda \right]}\rangle \langle {\vec{\omega }}_{\mu }^{\prime [\lambda ]}|\right)\\ & = & \sum_{i,j=0}^{d-1}|i\rangle \langle j|\mathrm{Tr}\left(\frac{1}{k}T_{ji}\sum_{\mu }|{\vec{\omega }}_{\mu }^{\left[\lambda \right]}\rangle \langle {\vec{\omega }}_{\mu }^{\prime [\lambda ]}|\right)\\ & = & \sum_{i,j=0}^{d-1}|i\rangle \langle j|\left(\frac{m_{\left[\lambda \right]}}{k}\langle {\vec{\omega }}^{\prime [\lambda ]}|T_{ji}|{\vec{\omega }}^{\left[\lambda \right]}\rangle \right), \end{array}$$

where $\langle {\vec{\omega }}^{\prime [\lambda ]}|T_{ji}|{\vec{\omega }}^{\left[\lambda \right]}\rangle$ is exactly the matrix element of irreducible representation corresponding to Young diagram [λ] for generator $T_{ji}$ in Lie algebra u(d) (do not worry about this part, the general matrix form of $T_{ji}$ in irreducible representation $D^{\left[\lambda \right]}$ of u(d) has been calculated by mathematicians and you can refer to [15]). Taking subsystem *A* into account, one can obtain

(22) $$\begin{array}{ccc} & & {\mathrm{Tr}}_{{\left(AB_{1}\right)}^{c}}\left(\rho_{AB_{1}B_{2}\cdots B_{k}}\right)\\ & = & \sum_{\left[\lambda \right]}\sum_{m,n=0}^{d_{A}-1}\sum_{i,j=0}^{d-1}|m\rangle \langle n|⊗|i\rangle \langle j|\\ & & \times \sum_{\vec{\omega },{\vec{\omega }}^{\prime }}\sigma {\left(\left[\lambda \right],\vec{\omega },\vec{\omega^{\prime }}\right)}_{mn}\frac{m_{\left[\lambda \right]}}{k}\langle {\vec{\omega }}^{\prime [\lambda ]}|T_{ji}|{\vec{\omega }}^{\left[\lambda \right]}\rangle f\left(\left[\lambda \right],\vec{\omega },\vec{\omega^{\prime }}\right). \end{array}$$

For every given [λ], the number of different values for $\vec{\omega }$ and $\vec{\omega^{\prime }}$ is just the dimension of the u(d)-irreducible representation $D^{\left[\lambda \right]}$. Therefore, ignoring the size of subsystem *A*, the size of the searching space in dealing with symmetric extension is given by

(23) ∑D[λ]2=d2−1+kk<d2k,

where the summation runs over all possible proper Young diagrams. One may conclude that the dimensions of the entire searching space grow no faster than $O(k^{d^{2}})$, which is significantly smaller than the original $O(d^{2k})$, therefore, the efficiency of SDP can be greatly improved when dealing with symmetric extension problems.

To guarantee the positive definiteness of the solved global matrix, one can test whether the density matrix corresponding to a different permutation is positive definite, and hence each testing needs far fewer resources.

To investigate the amount of resources needed for each single testing, one should focus on the growth rates of $D^{\left[\lambda \right]}$ and $m_{\left[\lambda \right]}$. The asymptotic behavior of the upper bound of $D^{\left[\lambda \right]}$ is given by

(24) $$\prod_{m=1}^{d-1}\frac{1}{(m)!}{\left(1+\frac{2k}{d(d+1)}\right)}^{\frac{d(d-1)}{2}},$$

which corresponds to the irreducible representation that satisfies $\lambda_{i}-\lambda_{i+1}\approx 2k/d\left(d-1\right)$ [16]. For a given *k*, the number of different valid Young diagrams whose number of rows is less than or equal to *d* is hard to compute analytically, but for a sufficiently large *k*, the asymptotic value is 1d!k+d−1k. To find a partition (not necessarily a partition that corresponds to a valid Young diagram!) that satisfies $\sum_{i}^{k}\lambda_{i}=k$ is equivalent to inserting d−1 separators between a line of *k* balls, which reads k+d−1k. Since some $\lambda_{i}$’s might be identical, the number of valid different Young diagrams should be less than 1d!k+d−1k, but when *k* is sufficiently large, the odds of any $\lambda_{i}$’s being identical approaches 0, so the asymptotic number of valid Young diagrams is given by 1d!k+d−1k.

## 4. Numerical Results

First, we apply our algorithm to the famous bipartite Werner state $\rho_{W,d}\left(\alpha \right)\in H_{d}⊗H_{d}$

$$\rho_{W,d}\left(\alpha \right)=\frac{1}{d^{2}-d\alpha }I-\frac{\alpha }{d^{2}-d\alpha }\sum_{ij}|ij\rangle \langle ji|,\;\alpha \in \left[-1,1\right].$$

Previous work [17] proved that the Werner state is (1,k)-extendible for $\alpha \in [-1,\frac{k+d^{2}-d}{kd+d-1}]$. As *k* goes to infinity, it gives the separable Werner state $\alpha \in \left[-1,\frac{1}{d}\right]$. To obtain such a (1,k)-extendible boundary $\alpha_{k}^{*}$, one can solve the following semi-definite programming

$$\begin{array}{cc} \; & \max\;c,\\ s.t. & \left\{\begin{array}{c} \rho_{AB_{1}\cdots B_{k}}⪰0,\\ \left(1^{A}⊗P_{ij}\right)\rho_{AB_{1}\cdots B_{k}}\left(1^{A}⊗P_{ij}\right)=\rho_{AB_{1}\cdots B_{k}},\\ {\mathrm{Tr}}_{B_{1}^{c}}\left(\rho_{AB_{1}\cdots B_{k}}\right)=\left(1-c\right)\rho_{o}+c\rho_{W,d}\left(1\right), \end{array}\right. \end{array}$$

where $\rho_{0}$ denotes the maximally mixed state (as semi-definite programming requires linear or affine equation constraints, we convert the non-linear expression α in the Werner state into a linear interpolation $\left(1-c\right)\rho_{0}+c\rho_{W,d}\left(1\right)$ used in the optimization), and the boundary can be calculated from the optimal value $\alpha_{k}^{*}=\frac{c^{*}d}{c^{*}+d-1}$.

The results are shown in Table 1. We compare the time required with the software QETLAB [18], a widely used MATLAB package in the quantum information community. The benchmark is performed on a standard laptop, AMD R7-5800H, 16 CPU cores (hyperthread enabled), 16 GB memory, and our algorithm is implemented in the CVXPY package [19] with the MOSEK solver [20]. The solved boundary $\alpha_{k}^{*}$ is within an absolute error of $10^{-8}$ compared with the analytical results. From the results, a significant speedup can be observed and a much larger *k*-extension problem can be handled by our algorithm.

We explicitly calculate the dimensions of the searching space and the number of parameters required to be tested for positive definiteness, which demonstrates the efficiency of our algorithm, as shown in Table 2 (our algorithm needs to undergo multiple different positive-definiteness tests, where each different Young diagram corresponds to its own test, but each individual test involves a significantly smaller matrix, and hence the efficiency is improved).

## 5. Discussion

The complexity of our new algorithm for dealing with k-symmetric extensions of quantum states is $O(k^{d^{2}})$, which is an improvement over the original algorithm with $O(d^{2k})$ complexity. However, it is important to note that the complexity of detecting entanglement is a QMA problem, which means that it is generally considered to be computationally hard. Although our new algorithm reduces the computational complexity of the problem, it does *not* change the fundamental difficulty of detecting entanglement. This is due to the fact that the size of the input of this problem is given by O(logk,d), and hence the resources needed in our algorithm still grow exponentially relative to the input. Therefore, while our algorithm presents some advance, it does not contradict the known fact that detecting entanglement is a QMA problem. The challenge of detecting entanglement remains an important area of research, with many open questions and opportunities for new breakthroughs.

## Figures and Tables

**Table 1:** Time usage for calculating the Werner (1,k)-extendible boundary. The dashed line “-” indicates the optimization failed due to memory limitation or intolerable time usage.

(d,k)	QETLAB (s)	irrep (s)	$\alpha_{k}^{*}$
(2,8)	0.19	0.16	0.588
(2,10)	12.60	0.16	0.571
(2,16)	-	0.32	0.545
(2,32)	-	3.18	0.523
(2,32)	-	51.96	0.512
(3,3)	0.62	0.51	0.818
(3,4)	7.96	2.38	0.714
(3,5)	-	11.56	0.647
(3,6)	-	55.60	0.6

**Table 2:** Dimensions and number of parameters needed in positive definiteness.

(d,k)	#Searching Space (QETLAB, irrep)	#Positive Definiteness (QETLAB, irrep)
(3,3)	(729,165)	$\left(729,\;10^{2}+8^{2}+1^{2}\right)$
(3,4)	(6561,495)	$\left(6561,\;15^{2}+15^{2}+6^{2}+3^{2}\right)$
(3,5)	(59,049, 1287)	(59,049, $21^{2}+24^{2}+15^{2}+6^{2}+3^{2})$
(3,6)	(531,441, 3003)	(531,441, $28^{2}+35^{2}+27^{2}+10^{2}+10^{2}+8^{2}+1^{2})$
(4,3)	(4096, 816)	$\left(4096,\;20^{2}+20^{2}+4^{2}\right)$
(4,4)	(65,536, 3876)	(65,536, $35^{2}+45^{2}+20^{2}+15^{2}+1^{2})$
(4,5)	(1,048,576, 15,504)	(1,048,576, $56^{2}+84^{2}+60^{2}+36^{2}+20^{2}+4^{2})$

## Data Availability

The authors agree with the availability of their research data.
